# A Novel CAF‐Related Signature for Precise Prediction of Clinical Outcomes and Immunotherapy Response for Breast Cancer Patients: Based on Multiomics Analyses and Experimental Validation

**DOI:** 10.1155/mi/9934495

**Published:** 2026-06-26

**Authors:** Qi Wang, Jing Chen, Shuyao Zhang, Yi Liu, Lian Xu, Lin Tian, Junqi Ren, Zhanhai Su, Zhengang Tang

**Affiliations:** ^1^ Department of Pathology, Renmin Hospital, Hubei University of Medicine, Shiyan, Hubei, China, hbmu.edu.cn; ^2^ Hubei Key Laboratory of Wudang Local Chinese Medicine Research, Hubei University of Medicine, Shiyan, Hubei, China, hbmu.edu.cn; ^3^ The Third Clinical College, Hubei University of Medicine, Shiyan, Hubei, China, hbmu.edu.cn; ^4^ Research Center for High Altitude Medicine, Qinghai University, Xining, Qinghai, China, qhu.edu.cn; ^5^ Department of Oncology, The Second Hospital of Dalian Medical University, Dalian, Liaoning, China, dlmedu.edu.cn; ^6^ Department of Pathology, The Second Affiliated Hospital of Guangdong Medical University, Zhanjiang, Guangdong, China, gdmu.edu.cn; ^7^ Neurological Disease Research Institute, Renmin Hospital of Hubei University of Medicine, Shiyan, Hubei, China, hbmu.edu.cn; ^8^ Shiyan Key Laboratory of Reproduction and Genetics (Renmin Hospital, Hubei University of Medicine), Shiyan, China; ^9^ Hubei Clinical Research Center for Reproductive Medicine, Shiyan, 442000, Hubei Province, China, hbmu.edu.cn

**Keywords:** breast cancer, cancer-associated fibroblasts, CD8^+^ t cell infiltration, single-cell RNA sequencing, tumor microenvironment

## Abstract

**Objective:**

Breast cancer (BC) remains a leading cause of cancer‐related mortality worldwide. Cancer‐associated fibroblasts (CAFs) is a central stromal component of the tumor microenvironment (TME), critically influence BC progression and therapeutic resistance. However, the association between CAF heterogeneity and patient prognosis or response to immunotherapy remains poorly characterized. Here, we aimed to develop a CAF‐associated gene signature by integrating single‐cell RNA sequencing (scRNA‐seq) and bulk RNA‐seq data to predict clinical outcomes and immunotherapeutic response in BC.

**Methods:**

Gene expression profiles and clinical data from BC patients were sourced from TCGA and GEO databases. scRNA‐seq data preprocessed (quality control, PCA, UMAP using Seurat) identified CAF‐related genes. Prognostic genes were identified via univariate Cox, lasso, and multivariate Cox regression. Single‐cell Gene Set Enrichment Analysis (scGSEA) assessed the signature’s link to immune infiltration and immunotherapy genes. R tools evaluated signature characteristics and real‐world applications. GO enrichment analysis was used to explore signaling pathways. CAF factor expression and CD8^+^ T‐cell correlation in clinical BC samples were validated using qPCR, immunohistochemical (IHC), multiplex immunofluorescence (mIF), and Western blot.

**Results:**

scRNA‐seq analysis identified multiple CAF‐specific marker genes that formed the core of our signature. Eight genes (ANXA5, APOD, CXCL14, GSN, IGFBP4, PPIB, TCF7L2, and TMEM98) were associated with favorable prognosis (low‐risk), whereas three genes (SDC1, EMP1, and FAM114A1) conferred higher risk. A risk score model based on these 11 genes independently predicted overall survival (OS) across diverse BC pathological subtypes, demonstrating robust prognostic accuracy. Immune infiltration analysis revealed significantly reduced immune cell abundance in the high‐risk group compared to the low‐risk group, suggesting diminished responsiveness to immunotherapy. In tumor tissues relative to adjacent nontumor tissues, mRNA and protein levels of the high‐risk genes (SDC1, EMP1, and FAM114A1) were consistently elevated (all *p* < 0.05). Moreover, both Western blotting and mIF showed significantly higher CAF abundance in high‐risk samples (*p* < 0.01), concomitant with markedly lower CD8^+^ tumor‐infiltrating lymphocyte counts (*p* < 0.05). GO enrichment analyses indicated that CAFs promote BC evolution and progression through complex signaling networks. Key pathways included extracellular matrix (ECM) remodeling, cell adhesion, tumor associated inflammation, and oncogenic cascades such as KRAS, WNT, IL‐6/STAT3, and TNFα/NF‐κB highlighting CAFs as pivotal regulators and potential therapeutic targets in BC.

**Conclusion:**

We present a novel CAF associated gene signature that robustly predicts prognosis and immunotherapy response in BC. As an independent prognostic indicator strongly correlated with immune infiltration, this model holds promise for guiding personalized therapeutic strategies. Future validation in large, multicenter cohorts and extension to other malignancies are warranted to facilitate clinical translation of CAF targeted biomarkers.

**Summary**

• We developed a novel CAF‐related gene signature by integrating single‐cell and bulk RNA‐seq data that effectively predicts survival outcomes and response to immunotherapy in breast cancer patients.
• Multiomics analyses revealed that cancer‐associated fibroblasts (CAFs) suppress CD8^+^ T cell infiltration via extracellular matrix remodeling and immunosuppressive signaling in the tumor microenvironment.
• We validated key CAF‐related genes in clinical samples, establishing a prognostic biomarker signature with strong translational potential for personalized therapy in breast cancer.

## 1. Introduction

Breast cancer (BC) is the most frequently diagnosed malignancy and a leading cause of cancer‐related death among women worldwide. The predominant histological subtype is invasive breast carcinoma (BRCA), which accounts for 75%–85% of all cases [1]. Despite substantial advances in early detection and systemic therapy, disease recurrence remains a major clinical challenge, particularly in advanced stages. Notably, the overall 5‐year survival rate for metastatic BC is ~20%, by contrast, exhibits 5‐year survival rates exceeding 90% [2, 3]. Epidemiological studies have linked rising incidence and mortality to modifiable risk factors, including chronic benign breast conditions, excessive alcohol consumption, metabolic syndrome, and increasing obesity prevalence [4]. Over the last few decades, advances in omics technologies have significantly improved the understanding of the molecular pathogenesis of BRCA [5, 6]. Molecular signatures derived from genomic, transcriptomic, and epigenomic data are now routinely used to stratify patients by risk and predict clinical outcomes [7]. Among these, multigene prognostic models hold particular promise for guiding personalized management in BRCA.

The tumor microenvironment (TME) comprises both malignant and nonmalignant stromal components. Historically, cancer cells were considered the sole drivers of tumorigenesis [8]. However, accumulating evidence demonstrates that reciprocal crosstalk between cancer cells and stromal elements critically fuels tumor progression, metastasis, and therapeutic resistance [9]. The stromal compartment includes fibroblasts, pericytes, mesenchymal stem cells (MSCs), immune cells, and a dynamic extracellular matrix (ECM) [10]. Central to this ecosystem are cancer‐associated fibroblasts (CAFs)—activated fibroblasts that can originate from resident tissue fibroblasts, bone marrow‐derived MSCs, hematopoietic stem cells, adipocytes, endothelial cells, or even through transdifferentiation of epithelial or cancer cells [11, 12]. CAFs are prevalent across diverse carcinomas, including breast and prostate cancers, and their protumorigenic interactions with malignant cells are well established [13–15]. CAFs promote tumor progression through multiple mechanisms: they secrete growth factors, cytokines, and chemokines; remodel the ECM via matrix metalloproteinases; and foster an immunosuppressive milieu—all of which enhance cancer cell proliferation, invasion, metastasis, and resistance to chemotherapy and targeted therapies [16–18]. Importantly, CAFs retain their tumor‐supportive phenotype even in the absence of direct contact with cancer cells, underscoring their autonomous role in shaping a permissive TME [19]. Consequently, targeting CAF‐derived signaling molecules or disrupting their downstream effector pathways has emerged as a promising therapeutic strategy in BRCA.

Despite extensive research on CAFs in breast cancer, their comprehensive molecular characterization and clinical relevance, particularly in relation to prognosis and response to immunotherapy, remain incompletely understood. In this study, we integrated publicly available single‐cell RNA sequencing (scRNA‐seq) and bulk transcriptomic datasets to dissect CAF heterogeneity in BRCA. We identified distinct CAF subpopulations and derived a CAF‐associated gene signature linked to patient risk. We further evaluated the clinical utility of this signature, its association with the immune landscape, and its predictive value for immunotherapy response. Finally, we developed a novel nomogram that integrates the CAF‐based risk score with key clinicopathological features to enhance prognostic accuracy. Our work provides new insights into CAF biology in BRCA and may facilitate the development of more precise, stroma‐informed therapeutic strategies.

## 2. Materials and Methods

### 2.1. Data Acquisition and Preprocessing

From the XENA website, select the TCGA GDC breast cancer (BRCA) data (https://xenabrowser.net/datapages/?cohort=GDC%20TCGA%20Breast%20Cancer%20BRCA) as the training set. The total number of samples was 1194, of which 1082 were cancer samples and 112 were standard samples. The validation set was downloaded from cBioportal as METABRIC (https://cbioportal-datahub.s3.amazonaws.com/brca_metabric.tar.gz), containing 1903 samples as shown in Table 1.

**Table 1:** Breast cancer database mRNA expression data set.

Sample type	TCGA data set	METABRIC
Normal	112	0
Tumor	1082	1903

Single‐cell sequencing data were used from the published BC study (GSE176078), which used 10x Genomics sequencing to generate single‐cell transcriptomes from 26 BC patients. Breast cancer drug resistance data were obtained from the GDSC database (https://www.cancerrxgene.org) and the CCLE database (https://portals.broadinstitute.org/ccle). Immunotherapy cohort data (IMvigor210) (http://researchpub.gene.com/IMvigor210CoreBiologies/packageVersions/). This study is the use of the urothelial carcinoma IMvigor210 cohort for immunotherapy validation. While this supports a conserved mechanism of stromal‐mediated immune exclusion, future validation in BC‐specific cohorts is required.

## 3. Analysis Methods

### 3.1. Single‐Cell RNA‐Seq Data Analysis

ScRNA‐seq data (GSE176078/GSE161529) were processed using Scanpy following standard quality control, normalization, dimensionality reduction (PCA), and clustering procedures. Cell types were annotated based on canonical marker genes and cross‐referenced with original study annotations.

### 3.2. Gene Function Analysis of CAFs Marker

GO enrichment analysis of CAF‐specific marker genes was performed using the R package clusterProfiler. Significantly enriched terms were defined by a threshold of *p*  < 0.05.

### 3.3. Prognostic Analysis of CAF Marker Genes

Patients were stratified into high‐ and low‐expression groups based on the median expression of each CAF marker gene. Kaplan–Meier (KM) survival curves were generated using the R packages survival and survminer, with log‐rank tests assessing significance.

### 3.4. CAF‐Related Signature

Using the coxph function in the R package ‘survival’, a univariate Cox regression model was constructed based on survival time and survival status to screen for CAFs subgroup genes associated with prognosis. Screening was conducted based on *p*‐values < 0.05, and then the R package ‘glmnet’ was used. The prognostic model (or classifier model) was constructed under 10‐fold cross‐validation multiplicity by the Cox method, the characteristic factors were screened, and the ROC curve of feature score and survival rate was built using the R package ‘pROC’. The training set was divided into two groups (high‐ and low‐risk) based on the optimal threshold.

### 3.5. CAF‐Related Signature Differences Between High and Low Groups in Different Clinical Characteristics

For different clinical characteristics, box‐and‐whisker plots were drawn for the high‐ and low‐group samples using the R package ‘ggplot2’ to visualize the distributions, and differences between groups were assessed using a *t*‐test.

### 3.6. CAF‐Related Signature: Independent Prognostic Factor Test

Univariate and multivariate Cox regression analyses were performed to evaluate whether the CAF‐derived risk score was an independent predictor of survival after adjusting for age, stage, grade, and other relevant clinicopathological variables (*p* < 0.05 considered significant).

### 3.7. CAF‐Related Signature Mutation Analysis

The R package ’maftools’ was used to compare samples from high‐ and low‐CAF‐related signature subgroups for differences in mutation levels. Fisher’s test was used with a threshold of *p*  < 0.05 to determine whether the top 20 genes differed significantly between the high and low subgroups.

### 3.8. CAF‐Related Signature CNV Information Display

The distribution of CAF‐related signature CNVs in the genome was plotted using the R package ‘ggplot2’.

### 3.9. CAF‐Related Signature Hallmark Pathway Enrichment Differential Analysis

Single‐sample gene set enrichment analysis (ssGSEA) was conducted using the GSVA package with the MSigDB hallmark gene sets. Pathway activity scores were compared between risk groups via *t*‐tests, and results were visualized as heatmaps using pheatmap.

### 3.10. CAF‐Related Signature Differences in Immune Characteristics

The immune score, matrix score, and evaluation score of each sample were evaluated based on the expression profile using the R package ‘estimate’. Then, the differences in these scores between the high‐feature group and the low‐feature group were plotted using the R package ‘ggplot2’, and the significance of the differences between them was calculated using the *t*‐test.

### 3.11. CAF‐Related Signature Differences in Immune Infiltration

The ‘IOBR’ R package was used to calculate the percentages of immune cell subsets in CIBERSORT, XCELL, and EPIC. The ‘ggplot2’ R package was used to create plots showing the score differences between the high‐ and low‐signature subgroups. A *t*‐test was used to assess the statistical significance of the differences.

### 3.12. CAF‐Related Signature Chemotherapy Resistance Analysis

Data for breast cancer drug resistance were downloaded from the GDSC database. The association between drug IC50 values and the gene signature was computed for each cell line using its gene expression matrix. With a threshold of *p* < 0.05 for statistical significance, drugs with significant differences in area under the curve (AUC) between the high and low subgroups were selected.

### 3.13. qPCR mRNA Analysis

To validate the expression of key CAF‐related genes, we performed RT‐qPCR on formalin‐fixed, paraffin‐embedded (FFPE) tumor and paired adjacent normal tissues from BC patients. Sample collection was approved by the Institutional Ethics Committee, and RNA integrity was preserved according to standard protocols. Detailed experimental procedures are provided in the Supporting Information.

### 3.14. Analysis of Protein Expression of CAF‐Related Factors in Clinical Samples

Protein expression of signature genes was first assessed using the Human Protein Atlas (HPA). For genes not covered in HPA, immunohistochemical (IHC) staining was performed on FFPE tissue sections from our cohort. Briefly, after deparaffinization and antigen retrieval, sections were incubated with primary antibodies against target CAF‐associated proteins. Signal detection was performed using DAB chromogen, and spatial localization was evaluated microscopically. Protein expression in CAFs versus normal stromal cells was semiquantified using the H‐score, which integrates staining intensity (0–3) and the percentage of positive cells (0%–100%), yielding a composite score ranging from 0 to 300. Subgroup analyses were conducted across ER^+^, HER2^+^, and TNBC subtypes to assess CAF heterogeneity.

### 3.15. High‐Dimensional Weighted Gene Coexpression Network Analysis (hdWGCNA) for CAFs

Four breast cancer samples from GSE161529 were selected for inclusion in the present analysis. Weighted gene coexpression network analysis (WGCNA) was performed using the ‘hdWGCNA’ R package, which is specifically designed for coexpression network analysis with small sample sizes. The analysis was conducted on the top 5000 most variable genes, ranked by variance, to focus on the most dynamically expressed transcripts. A soft‐power threshold was selected based on the scale‐free topology criterion, ensuring that a signed hybrid network was constructed that approximates a scale‐free topology. Subsequently, a topological overlap matrix (TOM) was computed to assess network interconnectedness, and genes were clustered using hierarchical clustering with average linkage. Dynamic tree cutting was applied to the resulting dendrogram to identify coexpression modules, which were assigned arbitrary color labels. The first principal component (module eigengene, ME) of each gene module was calculated to represent the overall expression pattern of the entire module. Relationships between modules were assessed by calculating the dissimilarity of their eigengenes and visualized via a hierarchical clustering dendrogram. Gene significance (GS) was defined as the correlation between individual gene expression and a key sample trait of interest. Module significance was subsequently evaluated as the average absolute gene significance for all genes within a module, identifying modules most strongly associated with the biological trait under investigation.

### 3.16. Statistical Methods

GraphPad Prism 10.0 software was used for all statistical analyses. Values have been presented as mean ± standard deviation of the mean (SEM). The paired *t*‐test, Mann–Whitney test, two‐tailed *χ* ^2^ test, and unpaired Student’s *t*‐test were utilized to contrast the two groups. In all cases, a *p*‐value < 0.05 was considered statistically significant. GraphPad 10, Citespace Advanced, SPSS 28.0, RStudio 2.4.1, and www.xiantaozi.com were used during the study for summarization, data mining, and statistical analysis. Mean ± standard deviation was used to depict data with normal distributions, and between‐group differences were analyzed using one‐way ANOVA. For variables with skewed distributions, the median and quartiles were used, and for multigroup comparisons, the rank‐sum test was used. *p* < 0.05 was statistically significant.

## 4. Results

### 4.1. Single‐Cell Data Analysis and CAFs Marker Gene Identification

**Figure 1:**
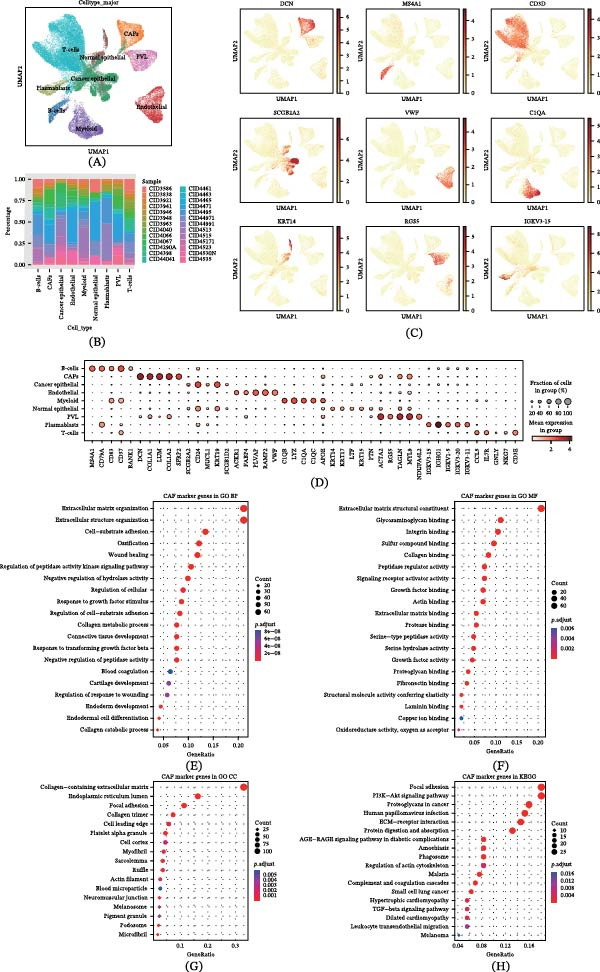
Single‐cell data analysis reveals marker genes and functional enrichment of CAFs in breast cancer. (A) UMAP of cell populations. (B) Sample distribution of cell populations. (C) Marker gene UMAP plot of different cell populations. (D) Marker gene point plot of cell populations. (E–H) CAF marker genes GO and KEGG enrichment analysis of biological processes (BP), molecular functions (MF), and cellular components (CC).

To dissect the cellular architecture of BC at single‐cell resolution, we reanalyzed the published scRNA‐seq data (GSE176078). Uniform manifold approximation and projection (UMAP) revealed distinct cellular clusters (Figure 1A). Sample composition across clusters was heterogeneous, as confirmed by bar plots of sample proportions per cell (Figure 1B). After stringent quality control—including removal of cells with mitochondrial gene expression >20%—we annotated major cell lineages using canonical marker genes (Figure 1C). CAFs were specifically identified based on established markers (ACTA2, FAP, and PDGFRB), and their unique transcriptional signature was extracted. Dot plots further visualized the top marker genes for each annotated population (Figure 1D).

### 4.2. CAFs Marker Gene Functional Analysis

To detect the functions of the CAFs marker genes, we used the R package ‘ClusterProfiler’ to analyze the functional enrichment of the CAFs marker genes. According to the significance threshold of *p*  < 0.05, we screened the enriched pathways and GO terms for display (Figure 1E–G). We observed that CAF marker genes were significantly enriched for functions related to the ECM and cell–cell connections.

### 4.3. CAF‐Related Signature Independent Prognostic Factor Test

To determine whether the CAF‐related signature is an independent prognostic factor for cancer, we performed one‐way and multivariate Cox analyses on the training and test set data using the R package ’coxph’. The results showed that the CAF‐related signature was an independent prognostic factor when the threshold was set to *p* < 0.05 (Figure 2A–D).

Figure 2A prognostic model was constructed based on CAF relative risk genes and validated for its independent predictive efficacy. (A,B) Single‐factor multivariate Cox analysis for training set data. (C,D) Single‐factor multivariate Cox analysis for test set data. (E) KM survival curves of CAFs marker genes with significant prognostic differences in the Top15 candidate genes. Development and validation of the CAF‐related signature. (F) One‐way Cox screening for prognosis‐related CAFs gene. (G) Diagram of the dynamic process of LASSO screening variables and the selection process of the cross‐validation parameter *λ*. (H) Genes and their model coefficients are used to construct the model.

**Figure:**
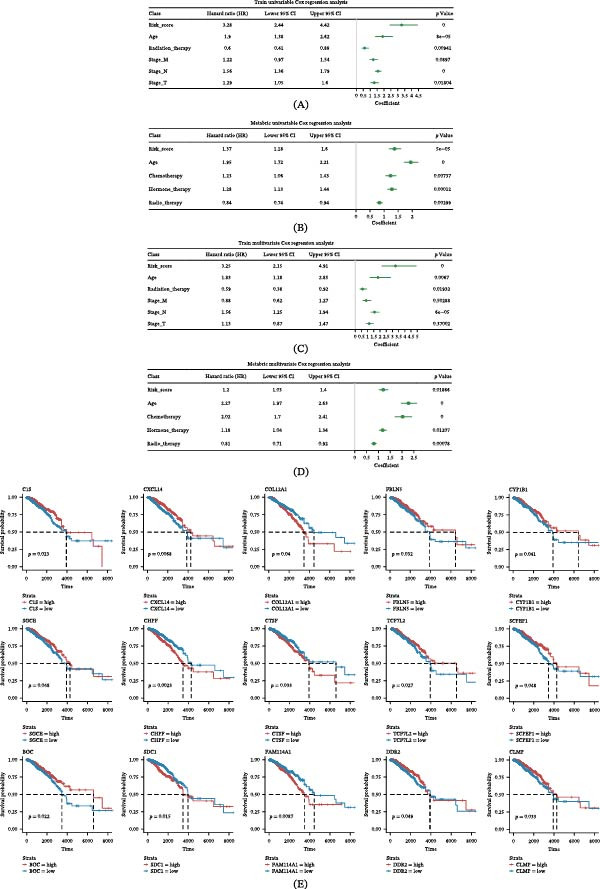

**Figure:**
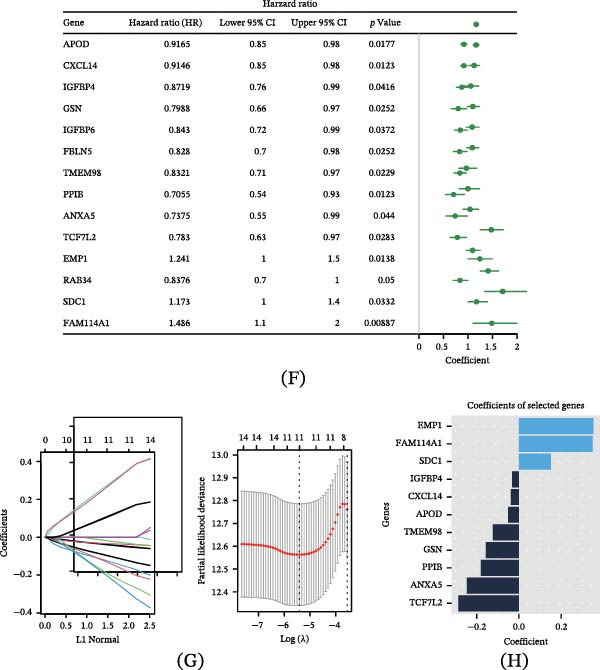

### 4.4. Effect of CAFs Marker Gene on Tumor Prognosis

To explore the effects of CAFs marker genes on tumor prognosis, we divided CAFs marker genes into high‐ and low‐expression groups based on median expression numbers. We then performed KM survival analyses using the R packages ’survival’ and ’survminer’ to determine whether there were differences in survival between high‐ and low‐expression groups. The following figure shows the top 15 KM survival curves for CAFs marker genes with significant prognostic differences (Figure 2E).

### 4.5. CAF‐Related Signature

To calculate the gene signature score profile for the CAF marker, we constructed a one‐factor Cox regression model based on survival time and status using the coxph function in the R survival package. We then screened to identify 14 prognostically relevant CAF subgroup genes with *p*‐values < 0.05 (Figure 2F). We then constructed a prognostic model using the ’glmnet’ R package and the Cox method with 10‐fold cross‐validation (Figure 2G) and screened the characterization factors. The genes and their corresponding model coefficients are shown in the attached table (Figure 2H). We then constructed an ROC curve of the signature score versus survival using the R package ’pROC’ and divided the training set into high‐ and low‐risk groups based on the optimal threshold.

### 4.6. The CAF‐Related Signature is Associated With Patients’ Prognosis and Clinical Characteristics

To test the CAF‐related signature with patient prognosis and also to test whether the CAF‐related signature can be equally applicable in external independent data METABRIC, we categorized the CAF‐related signature into high and low groups according to the same method to explore the CAF‐related signature and patient prognosis correlation OS. Using the R packages survival and survminer to perform KM survival curve analysis on the validation and training METABRIC sets, the results showed that the CAF‐related signature exhibited significant survival differences across subgroups (Figure 3A,D). Signature score distribution for training and validation sets and datais shown in (Figure 3B,E). The ROC curves and the distribution of signature scores showed that the model was reliable in both training and validation sets (Figure 3C,F).

Figure 3The high‐risk group was significantly associated with worse clinicopathological characteristics and specific mutational profiles. (A,D) KM survival curves for data from training and validation sets. (B,E) Signature score distribution for training and validation sets and data. (C,F), ROC curves for 1, 3, and 5‐year survival for training and validation sets and data. (G) Differences in K‐M survival analyses between high‐ and low‐risk groups in different clinical characteristics groups. (H) Differences of CAF‐related signature in clinical subgroups of training and test set data. (I) CNV frequency plot in the high‐risk or low‐risk group using ggplot2. (J) Violin plots comparing CNV frequency between high‐ and low‐risk groups, with significant difference indicated ( ^∗∗^ *p* < 0.01, *t*‐test). These results highlight distinct CNV patterns associated with CAF‐related risk groups. (K) CAF‐related signature mutations between high‐ and low‐risk groups. (L) Differences in hallmark enrichment between subgroups of the related signature (ns: *p* ≥ 0.05;  ^∗^: *p* < 0.05;  ^∗∗^: *p* < 0.01;  ^∗∗∗^: *p* < 0.001;  ^∗∗∗∗^: *p* < 0.0001).

**Figure:**
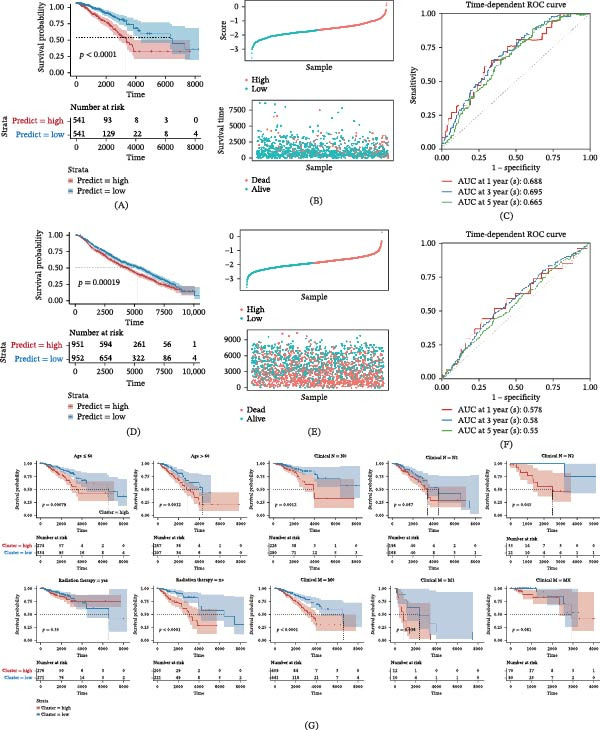

**Figure:**
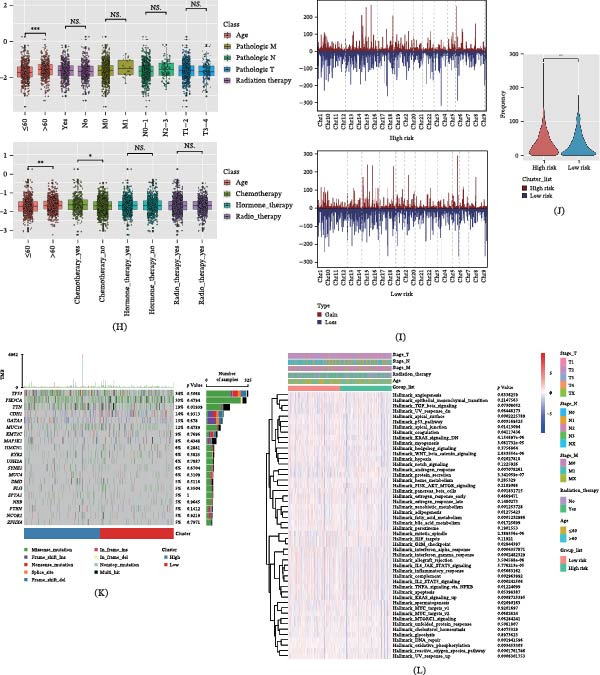

### 4.7. Analysis of CAF‐Related Signature Prognostic Efficacy by Subgroups With Different Clinical Characteristics

To test the stability of our CAF‐related features across different clinical features, we examined differences in survival status by age, radiotherapy, and pathological M and N features between the high‐ and low‐CAF‐related feature groups. The results revealed significant survival differences between the high‐ and low‐risk groups within almost all typical clinical subgroups (Figure 3G).

### 4.8. Signature Correlates With Patient Clinical Characteristics

To determine whether CAF‐related features are associated with multiple critical clinical features in cancer, we selected several typical clinical features. Statistical analysis revealed significant differences in CAF‐related features between the G2 and G3 grades, as well as between the pathological T stages T1−2 and T3−4, in both the training and test sets (*p* < 0.05; Figure 3H).

### 4.9. CAF‐Related Signature, High‐ and Low‐Risk Group CNV Information Presentation

In this study, we explored the relationship between high‐ and low‐CAF‐related signature groups and the frequency of copy number variation. We used the R package ’ggplot2’ to plot the copy number variation frequency in high‐ and low‐CAF‐related signature groups (Figure 3I). Then, we used the *t*‐test to analyze the copy number frequency distribution between the high‐ and low‐CAF‐related feature groups and found significant differences (Figure 3J).

### 4.10. Differences in Mutations in High‐ and Low‐Risk Groups for CAF‐Related Signature

To explore the mutation differences between the high‐risk and low‐risk groups of CAF‐related characteristics, we used the R package ‘maftools’ to compare the mutation levels in the samples of the high‐risk and low‐risk subgroups of CAF‐related characteristics. We selected the top 20 mutant genes and used the Fisher test to detect differences in CAF‐related characteristics between the high‐ and low‐risk groups for each gene. We found significant differences in TTN between the high‐risk and low‐risk groups with CAF‐related traits (Figure 3K).

### 4.11. Pathway Differences Between High‐ and Low‐Risk Groups for the CAF‐Related Signature

ssGSEA of hallmark pathways showed that high‐risk tumors were enriched in epithelial‐mesenchymal transition (EMT), TNF‐α signaling, and inflammatory response, while low‐risk tumors exhibited stronger oxidative phosphorylation and estrogen response signatures (Figure 3L).

### 4.12. CAF‐Related Signature Assessment of Immunotherapy Prognosis

To test whether the CAF‐related signature can predict immunotherapy prognosis, we applied our risk score model to the IMvigor210 cohort. Then, we tested KM survival status between the high‐ and low‐risk groups. The KM survival curves across the subgroups differed significantly (Figure 4A). We then performed a statistical test on the distribution of CR/PR and PD/SD between the high‐ and low‐risk groups and found that the percentage of different response statuses between the two groups was not significant (Figure 4B; *χ*‐square test, *p* = 0.4351). Additionally, the risk scores across response subgroups did not differ significantly (Figure 4C). Our model was also found to be reliable in immunotherapy datasets, as indicated by the ROC curves and the distribution of signature scores (Figure 4D,E).

Figure 4The CAF‐related signature showed that the high‐risk group exhibits an immunosuppressive and poorer response to chemotherapy. (A) Kaplan–Meier survival curves showing significant differences in overall survival between high‐risk and low‐risk groups; (B) Bar chart depicting the distribution of response statuses (CR/PR vs. PD/SD) between high‐ and low‐risk groups (*p* = 0.4351, *χ*‐square test); (C) Box plot comparing risk scores across response subgroups; (D) Waterfall plot displaying the distribution of risk scores reordered by response status; (E) Time‐dependent ROC curve analysis demonstrating the predictive performance of the CAF‐related signature at 1, 3, and 5 years with corresponding AUC values. (F) IC50 lollipop plot of five drugs significantly associated with CAF‐related signature in the GDSC database. (G) IC50 lollipop plot of five drugs significantly associated with CAF‐related signature in the CCLE database. (H) AUC distribution of five drugs with significant differences in AUC between groups in the GDSC database. (I) AUC distribution of five drugs with significant intergroup AUC differences in the CCLE database. (J) Distribution of immunization score, matrix score, and Estimate Score among CAF‐related signature subgroups. (K) Correlation between CAF‐related signature and immunization score, matrix score, and Estimate Score. (L) Difference in CIBERSORT score between CAF‐related signature group differences in CIBERSORT score (ns: *p* ≥ 0.05;  ^∗^: *p* < 0.05;  ^∗∗^: *p* < 0.01;  ^∗∗∗^: *p* < 0.001;  ^∗∗∗∗^: *p* < 0.0001).

**Figure:**
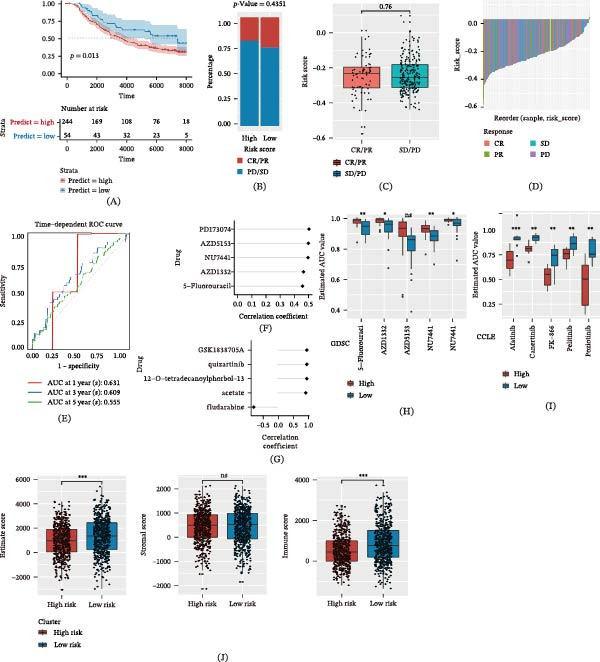

**Figure:**
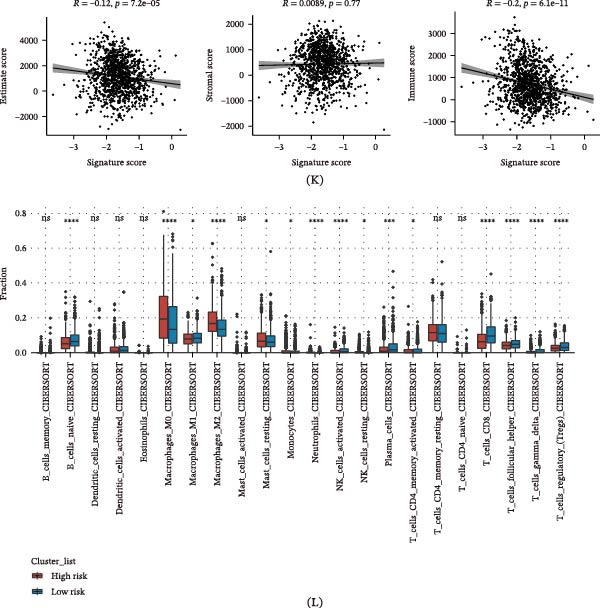

### 4.13. CAF‐Related Signature Intergroup Chemotherapy Resistance

To detect the differences in chemotherapy resistance between the high CAF‐related characteristic group and the low CAF‐related characteristic group, we downloaded breast cancer drug resistance data from GDSC and CCLE. We calculated the CAF‐related characteristics of the drug resistance test cell lines using the expression matrix of each cell line. It was also found that AZD5153, NU7441, PD173074, 5‐fluorouracil, and PD173074 showed the greatest resistance to treatment in the GDSC database. In the GDSC database, the CAF‐related signature showed a significant correlation with drug IC50 values (Figure 4F). In the CCLE database, the CAF‐related signature was significantly correlated with the IC50 of drugs when stimulated by the top five significantly related drugs: 12‐*O*‐tetradecanoylphorbol‐13‐acetate, quizartinib, GSK1838705A, carvedilol, and fludarabine (Figure 4G). We also examined whether there was any difference in the AUC between the high and low groups. In the GDSC database, the AUC values for four of the five drugs mentioned above showed significant differences between the high and low groups (Figure 4H). In the CCLE database, however, the difference in the top five drugs’ AUC values was not significant. We identified the drugs with substantial differences in the top five AUC values (Figure 4I).

### 4.14. Differences in Immune Characteristics Between High‐ and Low‐Risk Groups for the CAF‐Related Signature

To detect the differences in immune characteristics among different subgroups of CAF‐related features, we first used the R package ‘ESTIMATE’ to evaluate the immune score, matrix score, and estimated score of each sample based on the expression profile. Then, we used the R package ‘ggplot2’ to identify the differences in immune scores, matrix scores, and estimated scores among subgroups of CAF‐related features (Figure 4J). Ultimately, we utilized the R software package ‘ggplot2’ to plot the correlation graphs between the CAF‐related feature scores of each sample and the immune scores, matrix scores, and estimated scores (Figure 4K). We found that the feature scores were significantly correlated with all three scores. We used the R package ‘IOBR’ to calculate the proportion of immune cells in CIBERSORT, XCELL, and EPIC, respectively. Among the immune cell proportions calculated by CIBERSORT, there were statistically significant differences between subgroups for multiple cell types (Figure 4L). Similarly, the proportions of immune cells calculated by EPIC and XCELL also showed statistically significant differences between subgroups (Figures S1 and S2).

### 4.15. CAFs Gene, mRNA, and Protein Expression Levels in Clinical Samples

**Figure 5:**
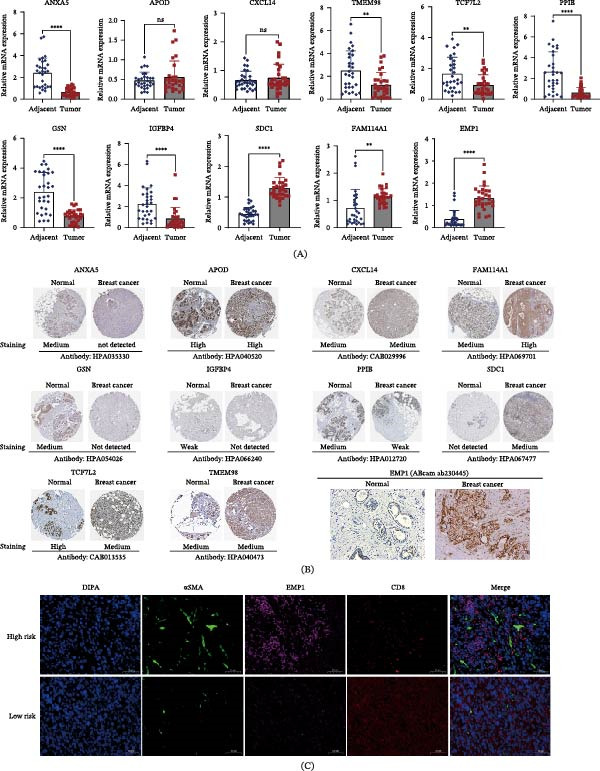
High‐risk genes exhibit significantly elevated mRNA/protein expression levels in cancerous tissues, inhibit CD8^+^ T cell infiltration, and colocalize with CAF markers. (A) The mRNA expression level of the key genes in the clinical samples. (B) The expression of key associated proteins in immunohistochemistry analysis. (C) Immunofluorescent staining showed the presence of SMA, EMP1, and CD8 in BC tumors (ns: *p* ≥ 0.05;  ^∗∗^: *p* < 0.01;  ^∗∗∗∗^: *p* < 0.0001).

The expression levels of 11 CAF‐related genes were analyzed in 30 paired samples (tumor and adjacent normal tissue) in BC to explore the clinical significance of these characteristics. qRT‐PCR results revealed that the mRNA expression levels of FAM114A1, SDC1, and EMP1 were higher in tumor samples and lower in standard tissue samples. There was no difference in the mRNA expression of APOD and CXCL14, while the expression levels of other mRNAs showed an opposite trend: lower in tumor samples and higher in standard tissue samples (Figure 5A). To investigate the expression of key proteins, we used the HPA database and our clinical patient samples to examine immunohistochemical staining of normal and tumor breast tissues (*n* = 6). EMP1 expression in cancer tissues was significantly higher than that in adjacent normal tissues. Except for TMEM98 and IGFBP4, the remaining proteins were consistent with the TCGA database and qRT‐PCR results (*p* < 0.05; Figure 5B). Considering the coefficients of the formulae, we inferred that the upregulation of EMP1, FAM114A1, and SDC1 and the downregulation of GSN, TCF7L2, ANXA5, and PPIB might have a combined effect on increasing the risk of tumorigenesis and poor prognosis.

### 4.16. CAFs’ Risk Factors in the TME are Negatively Correlated With the Level of CD8^+^ Immune Cell Infiltration

Multiplex immunofluorescence (mIF) on clinical specimens revealed a strong positive correlation between EMP1 and αSMA (CAF marker; *r* = 0.78, *p* < 0.01; Figure 5C), and a significant negative correlation between EMP1 and CD8^+^ T cell density (*r* = −0.65, *p* < 0.05). Western blotting further confirmed higher αSMA levels in EMP1‐high regions, concomitant with reduced CD8^+^ T cells (Figure S3), supporting a model wherein EMP1^+^ CAFs foster an immunosuppressive niche. Consistent with these findings, analysis of the TCGA‐BRCA cohort further confirmed a robust negative correlation between EMP1 mRNA levels and CD8+ T cell infiltration estimates (Spearman’s *r* = −0.42, *p*  < 0.001), suggesting that EMP1+ CAFs contribute to an immune‐excluded phenotype by physically or chemically restricting T‐cell access to tumor nests [18, 20]. These results were obtained from a relatively small cohort and should be interpreted as preliminary proof‐of‐concept evidence supporting our computational findings. Future studies using larger multicenter clinical cohorts are essential to robustly validate the prognostic and predictive value of the EMP1 signature before its potential translation into clinical practice.

### 4.17. hdWGCNA Identified the EMP1^+^ CAFs Core Gene Module and Coexpression Network

To explore the coexpressed gene network and hub genes in EMP1^+^ CAFs, we employed hdWGCNA. The analysis was performed on a high‐quality dataset of 10,655 cells derived from four BC samples of the GSE161529 dataset (Figure 6A). The UMAP visualization effectively captured cellular heterogeneity, and the expression distribution of the CAF marker gene COL1A1 is shown in Figure 6B. CAFs were distinguished from non‐CAF cells as shown in Figure 6C, and within CAFs, EMP1‐positive and EMP1‐negative cells were identified (Figure 6D). The optimal soft power threshold for hdWGCNA was determined to be *β* = 13 through scale‐free topology model fit analysis, as shown in Figure 6E. The hdWGCNA dendrogram in Figure 6F revealed nine distinct modules, each represented by a unique color. The kME and hME values for these modules across EMP1‐positive and EMP1‐negative CAFs are presented in Figure 6G,H, respectively. The M7 module exhibited the highest kME and hME values in EMP1‐positive CAFs, indicating a strong association with EMP1 expression. A dotplot in Figure 6I illustrated the correlation between the nine modules and EMP1‐positive and negative status, with the M7 module showing the strongest positive correlation with EMP1‐positive status. The gene coexpression network within the M7 module is depicted to highlight gene connectivity (Figure 6J). Figure 6K provided a comprehensive view of the coexpression network across all modules and hub genes, highlighting the complex interplay among cellular components. These results underscore the importance of the M7 module in EMP1‐positive CAFs and suggest that its genes may play a critical role in CAF biology, offering valuable insights into the molecular mechanisms underlying CAF functional heterogeneity in breast cancer.

Figure 6hdWGCNA identified the EMP1^+^ CAFs core gene module and coexpression network. (A) UMAP visualization of 10,655 high‐quality cells from four breast cancer (BC) samples of the GSE161529 dataset. (B) Expression distribution of the CAF marker gene COL1A1 across the cell clusters. (C) Distinction between CAFs and non‐CAF cells based on gene expression profiles. (D) Identification of EMP1‐positive and EMP1‐negative cells within the CAF population. (E) Determination of the optimal soft power threshold (*β* = 13) for hdWGCNA through scale‐free topology model fit analysis. (F) Dendrogram from hdWGCNA revealing nine distinct modules, each color‐coded for clarity. (G) kME values for the nine modules across EMP1‐positive and EMP1‐negative CAFs. (H) hME values for the nine modules across EMP1‐positive and EMP1‐negative CAFs. (I) The dotplot depicts the correlation between the nine modules and EMP1‐positive and negative status, with the M7 module showing the strongest positive correlation. (J) Gene coexpression network within the M7 module, and highlights the interconnectedness of genes associated with EMP1‐positive CAFs. (K) A comprehensive coexpression network across all modules and hub genes highlights the complex interplay among cellular components in EMP1‐positive CAFs.

**Figure:**
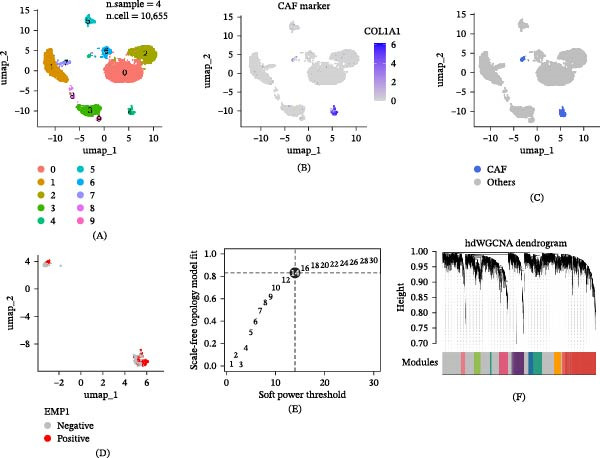

**Figure:**
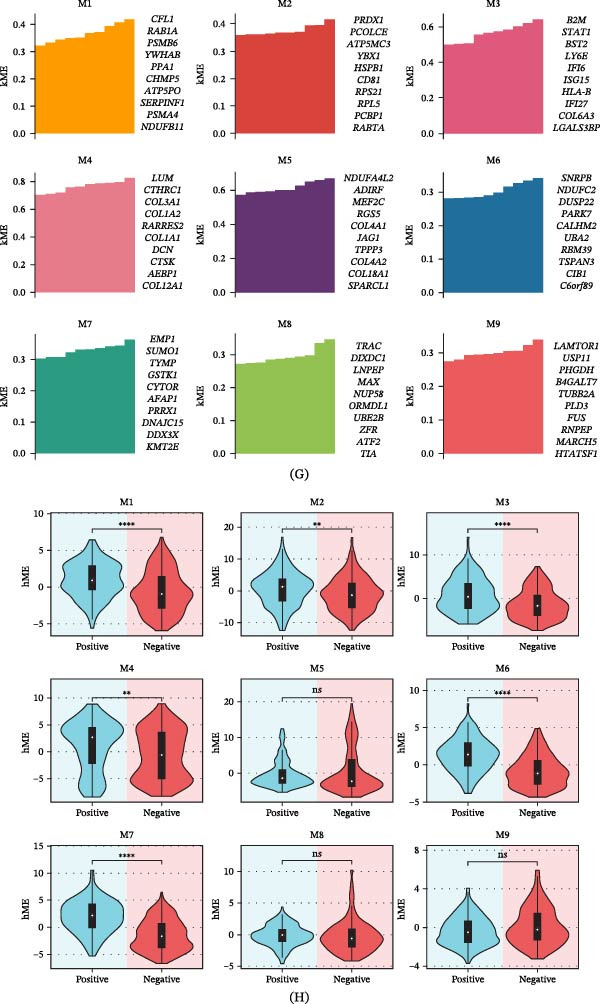

**Figure:**
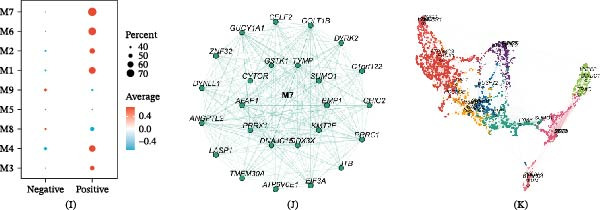

## 5. Discussion

In this study, we systematically characterized the landscape of CAFs in BC and developed an 11‐gene CAF‐related signature that independently predicts patient prognosis and response to immunotherapy. Our core finding identifies EMP1 as a critical hub gene within this signature, driving an immunosuppressive tumor microenvironment characterized by reduced CD8^+^ T‐cell infiltration. By integrating single‐cell resolution data with bulk transcriptomics and clinical validation, we provide compelling evidence that targeting the EMP1^+^ CAF subset could serve as a novel strategy to sensitize resistant breast cancers to immune checkpoint blockade. underscoring the central role of CAFs in shaping the TME.

We identified an 11‐gene CAF signature—comprising ANXA5, APOD, CXCL14, GSN, IGFBP4, PPIB, TCF7L2, TMEM98, SDC1, EMP1, and FAM114A1—that collectively reflects the functional duality of CAFs: while some genes (ANXA5, APOD, CXCL14, GSN, etc.) act as tumor suppressors, others (SDC1, EMP1, and FAM114A1) promote malignancy. This aligns with emerging evidence that CAFs are not a uniform population but rather a heterogeneous ensemble of functionally distinct subsets [21–23] In the present study, the integration of single‐cell and bulk RNA‐seq data further enhanced the characterization of CAFs features, enabling the construction of a more robust prognostic model. In contrast to previous approaches, ours also demonstrated reproducibility and reliability across different datasets and consequently suggests wider clinical applicability.

When compared to established prognostic tools such as Oncotype DX, MammaPrint, and PAM50, our CAF‐related signature offers distinct advantages. While current commercial assays primarily focus on tumor‐intrinsic proliferation and hormone signaling pathways, they often lack sensitivity in predicting immunotherapy response, particularly in TNBC. Our signature, by capturing the stromal component, provides independent prognostic value and superior stratification for immunotherapy benefit. Furthermore, the concise 11‐gene panel is amenable to detection via routine RT‐qPCR or IHC, presenting a more cost‐effective and widely accessible alternative for clinical implementation in resource‐limited settings compared to complex multigene genomic arrays [24, 25].

In BC research, gene expression levels are closely associated with patient prognosis. Analyzing the expression patterns of these genes can reveal their mechanisms of action in breast cancer development and provide new potential targets and therapeutic strategies to enhance clinical outcomes. Studies have shown that Annexin A5 (ANXA5) plays a crucial role in regulating apoptosis and antiinflammatory responses. Specifically, low expression of ANXA5 is particularly associated with poor prognosis in BC patients [26]. Insufficient ANXA5 expression may contribute to tumor progression by inhibiting apoptosis and suppressing tumor‐related inflammatory responses, thereby enhancing the proliferation and survival of breast cancer cells.

Apolipoprotein D (APOD) plays a role in lipid metabolism and antioxidant defense responses. This report illustrated a statistically significant association between reduced expression of APOD and poor prognosis in BC patients. Insufficient expression may contribute to tumor cell proliferation and malignant transformation by influencing lipid metabolism pathways and altering the composition and function of cell membranes [27]. CXCL14 is a chemokine closely associated with the infiltration of immune cells into tissuess [28]. In the current study, low CXCL14 expression in breast cancer was correlated with adverse clinical outcomes. Moreover, diminished CXCL14 levels may foster tumor growth and metastasis by suppressing the recruitment of key immune effectors—including T cells and macrophages—into the tumor microenvironment (TME), thereby attenuating antitumor immunity and facilitating immune escape from surveillance and destruction.

Gelsolin (GSN), a protein involved in cytoskeletal remodeling that governs cancer cell motility and metastasis, is significantly downregulated in patients with poor prognosis [29]. Reduced GSN expression compromises cytoskeletal stability, enhances tumor cell invasiveness, and promotes both local invasion and distant metastasis [30]. Similarly, low expression of insulin‐like growth factor binding protein 4 (IGFBP4) is strongly correlated with poor prognosis in BC patients [31]. IGFBP4 blocks growth and induces apoptosis by inhibiting the overactivation of insulin‐like growth factor, which otherwise stimulates cancer cell growth and survival. The low expression of another protein, peptidylprolyl isomerase B (PPIB), involved in protein folding and immune system regulation, also indicates a worse prognosis and is likely due to protein folding disorders and consequent cellular stress responses that lead to cancer progression [29]. TCF7L2 is an essential factor in the Wnt signaling pathway, and its reduced expression leads to inappropriate pathway activation, thereby enhancing the proliferation and survival of cancer cells, which promotes tumor growth [32, 33]. The reduced expression of transmembrane protein TMEM98 is also a negative regulator of FRAT‐mediated Wnt/ß‐catenin signaling. It has been implicated in various pathological conditions, including its association with worse prognosis [34].

In contrast, high expression of SDC1, a cell‐surface proteoglycan that plays a role in cell‐matrix interactions, is associated with a poor prognosis by facilitating tumor cell adhesion and migration within the extracellular matrix [35]. Elevated expression of EMP1, affecting growth, proliferation, and invasion, is also associated with poor outcome [36, 37]. It has been the subject of various studies in the context of breast cancer, with findings indicating both high‐ and low‐expression levels. This discrepancy in expression patterns suggests a complex role for EMP1 in BC pathogenesis. EMP1 was found to be expressed in over 90% of all invasive ductal carcinoma (IDC) subtypes, indicating a prevalent role in these cancer types. However, the study did not find a significant difference in overall survival (OS) or disease‐free survival based on EMP expression, suggesting that while EMP1 is widely expressed, its impact on prognosis may be limited or influenced by other factors [38, 39]. High expression of FAM114A1, a gene whose role in cell cycle regulation remains incompletely defined, is also linked to poor prognosis. Overexpression of FAM114A1 may drive tumorigenesis by accelerating cell cycle progression and enhancing proliferative capacity [40]. Emerging evidence from gastric cancer suggests a potential immunomodulatory function of FAM114A1 within immune cells of the TME, highlighting an avenue for future investigation in the development of novel therapeutic modalities.

Beyond breast cancer, EMP1 exhibits context‐dependent roles in other solid tumors. Especially in glioblastoma and lung cancer, EMP1 is considered a tumor promoter, primarily functioning through activation of the PI3K/AKT signaling pathway. In glioblastoma, studies have shown that EMP1 expression levels are closely associated with tumor malignancy and patient prognosis. EMP1 is a novel cell‐surface biomarker associated with acquired clinical resistance to the EGFR inhibitor gefitinib in lung adenocarcinoma. Suppressing EMP1 expression can significantly inhibit tumor cell proliferation and invasion, a process closely linked to the activation of the PI3K/AKT/mTOR signaling pathway [41, 42]. Additionally, EMP1 influences the stemness of glioma stem cells by regulating CD44 expression, providing a novel strategy for glioma treatment [43]. Furthermore, our data suggest a novel role for EMP1 in shaping the (TME, potentially by facilitating ECM remodeling that creates a physical barrier against immune cell infiltration. Given its stable membrane localization, EMP1 represents a promising candidate for targeted therapies, including antibody‐drug conjugates (ADCs), offering a potential avenue to overcome therapeutic resistance. Regarding targeted therapy, although no direct small‐molecule inhibitors specifically targeting EMP1 are currently in clinical trials, its membrane localization makes it an attractive candidate for ADCs or CAR‐T cell therapies, similar to successful strategies targeting other membrane proteins like Trop‐2 or HER2. We have discussed the prospect of developing EMP1‐targeted biologics and highlighted recent advances in a promising future direction.

Collectively, these findings demonstrate that the expression levels of this CAF‐related gene set are significantly correlated with BC progression and patient prognosis. An integrated understanding of their biological functions and regulatory networks provides a valuable framework for improving diagnosis, risk stratification, and the design of personalized treatment strategies. Such insights may ultimately enable more precise clinical management and enhance therapeutic efficacy in breast cancer patients. Beyond their roles in regulating tumor cell proliferation and metastasis, these hub genes also modulate the tumor immune landscape and angiogenesis‐related processes. For instance, in BC tissues, high expression of CXCL14 and GSN indirectly influences the infiltration of immune cells—particularly macrophages and dendritic cells and orchestrates cascades of tumor‐promoting signals during disease progression [44–46]. APOD, while primarily linked to lipid homeostasis, also contributes to cytoprotection through its antioxidant properties. It may thus help shape the TME by mitigating oxidative stress and indirectly participating in inflammatory and immune responses [47, 48]. Clinical samples confirmed that SDC1 and EMP1 overexpression enhances the role of fibroblasts surrounding cancer cells in vivo and CAFs expression in tumor tissues during tumor progression. In this study, we found that CAF‐related genes were mainly highly expressed in extracellular regions, in cell membrane binding and dependence, and in cell signaling pathways.

These results underscore the multifaceted roles of CAFs in tumor development. Pathway enrichment analysis indicated significant enrichment of CAF‐associated genes in biological processes including ECM–receptor interaction, contractile function, RhoA activation, interferon‐*α*/*γ* response, inflammatory response, and TGF‐*β* receptor signaling—all of which converge on Rac pathway activation. This signaling axis has been extensively implicated in cancer cell invasion and immune cell trafficking, both of which are tightly linked to clinical prognosis. Notably, immune infiltration analysis revealed a marked reduction in CD8^+^ T‐cell abundance in patients with high‐risk CAF signatures. This finding suggests that CAFs may impair responses to immunotherapy by fostering an immunosuppressive TME. Single‐cell RNA sequencing (scRNA‐seq) further enabled a high‐resolution dissection of CAF heterogeneity within the breast cancer microenvironment. UMAP‐based clustering showed consistent CAF populations across multiple patient samples, indicating conserved CAF‐specific gene programs in human breast tumors. Moreover, beyond GO signaling annotations, several CAF markers identified via single‐cell profiling were corroborated in public datasets and validated by clinical immunohistochemistry, reinforcing their reliability as prognostic indicators.

The genetic features associated with CAFs thus offer a promising tool for individualized patient assessment and open new avenues for stroma‐targeted, personalized therapies. Importantly, CAF‐related gene signatures correlate with response to immune checkpoint inhibitor therapy: patients in the high‐risk group exhibited significantly lower CD8^+^ T cell infiltration, implying that CAFs may indirectly suppress checkpoint inhibitor efficacy within the TME [49]. These observations align with findings in other malignancies, further establishing CAFs as a viable therapeutic target to overcome immunotherapy resistance.

Targeting CAF‐related pathways represents a promising therapeutic avenue. Emerging strategies include (1) inhibiting ECM remodeling enzymes such as LOX or FAP to normalize the stromal barrier and improve drug penetration [50]; (2) blocking key signaling axes like TGF‐*β* or IL‐6/JAK/STAT that drive CAF activation and immune suppression [51]; and (3) developing combination regimens that pair stroma‐targeting agents with ICIs. Specifically, depleting or reprogramming the EMP1^+^ CAF subset identified herein could potentially convert ’cold’ tumors into ’hot’ ones, thereby sensitizing resistant TNBC patients to immunotherapy.

Future perspectives: Modeling EMP1^+^ CAF–tumor crosstalk. To further elucidate the mechanistic underpinnings of EMP1^+^ CAF function, our future work will prioritize the establishment of patient‐derived 3D coculture systems and organoids. These models will incorporate EMP1^+^ CAFs, autologous tumor cells, and immune components to faithfully simulate the spatial architecture and molecular crosstalk of the TME. Such advanced in vitro platforms are essential for dissecting paracrine signaling loops—such as EMP1‐mediated ECM stiffening—that drive immune exclusion and therapeutic resistance, phenomena that are often lost in traditional 2D monocultures [52]. These TME mimics will serve as a robust preclinical screen for identifying effective stroma‐targeting combination therapies prior to validation in patient‐derived xenograft (PDX) models.

It is important to consider the inherent heterogeneity of CAFs. Recent single‐cell studies have delineated distinct CAF subpopulations, primarily myofibroblastic CAFs (myCAFs) and inflammatory CAFs (iCAFs), each exerting unique functions within the TME. Our identified EMP1^+^ CAF cluster exhibits high expression of contractile marker ACTA2, aligning it closely with the myCAF phenotype, which is traditionally associated with ECM deposition and physical restriction of T cells [9]. However, the unique transcriptional signature of EMP1^+^ CAFs suggests that they may represent a specialized subset distinct from classical myCAFs or iCAFs. This specificity might explain their potent association with immune exclusion, distinguishing them from iCAFs that primarily mediate suppression via cytokine secretion.

Although this study revealed the critical role of characterizing CAF‐related genes in BC, there are still some limitations. Some recent studies have highlighted the importance of CAFs in tumor progression and therapy resistance [53, 54]. First, due to the relatively small sample size, the model’s generalization performance needs to be validated in a larger, independent cohort. Second, the characterization of CAFs and their molecular mechanisms of action in different breast cancer subtypes has not been thoroughly investigated and requires further clarification through experimental validation. Furthermore, the mechanism by which CAFs regulate immune cell infiltration, particularly in the context of immunotherapy, remains poorly understood. Future work should prioritize (1) defining CAF subset‐specific roles in different BC subtypes using spatial transcriptomics and lineage tracing; (2) testing CAF‐targeting strategies (e.g., FAP inhibition and TGF‐*β* blockade) in combination with immunotherapy or chemotherapy in preclinical models; and (3) integrating our signature with imaging or liquid biopsy data to enable noninvasive risk stratification.

## 6. Conclusion

In summary, we present a clinically actionable CAF‐derived gene signature that integrates stromal biology with patient prognosis and treatment response in breast cancer. This signature not only serves as an independent predictor of survival but also reveals mechanistic links between CAF activity, ECM remodeling, and immunosuppression. Experimental validation confirms that high‐risk genes (SDC1, EMP1, and FAM114A1) are upregulated in tumor‐associated fibroblasts and correlate with reduced CD8^+^ T cell infiltration, positioning CAFs as key modulators of the immunotherapeutic landscape. While further validation in prospective cohorts and diverse cancer types is warranted, our findings offer a promising framework for stroma‐informed precision oncology—enabling risk‐adapted therapy selection and the rational design of CAF‐targeted combination regimens.

## Author Contributions

Zhengang Tang, Zhanhai Su, and Junqi Ren conceived and designed the work and drafted the manuscript. Qi Wang and Shuyao Zhang were responsible for data analysis and investigation. Qi Wang and Jing Chen performed sample staining experiments. Yi Liu, Lian Xu, and Lin Tian were responsible for editing the manuscript.

## Funding

This study was supported by the Key Research Project of Graduate Education at Hubei University of Medicine (Grant YJ202514), Shiyan City Guiding Research Project (Grant 24Y085), Research Projects of Shiyan People’s Hospital (Grant syrmyn‐2026‐015), Hubei Key Laboratory of Wudang Local Chinese Medicine Research, Hubei University of Medicine (Grant WDCM2026006), and Joint Fund Project of Hubei Natural Science Foundation (Grant 2025AFD200).

## Disclosure

All authors agreed on the manuscript and contributed to manuscript drafts and figure preparation.

## Ethics Statement

This study is a retrospective study on pathologically preserved specimens and does not involve clinical trials. It was approved by the Ethics Committee of Renmin Hospital, Hubei University of Medicine (Number SYRRMYY2022‐042), conducted in accordance with local legislation and institutional requirements, and followed relevant ethical principles, including those of the Declaration of Helsinki.

## Consent

Informed consent was secured from all participants.

## Conflicts of Interest

The authors declare no conflicts of interest.

## Supporting Information

Additional supporting information can be found online in the Supporting Information section.

## Supporting information


**Supporting Information** Figure S1: The proportion of immune cells across groups was calculated using EPIC. Figure S2: XCELL calculated immune cell ratios between different groups. Figure S3: CAFs’ risk factors are negatively correlated with the level of CD8+ immune cell infiltration. The expression was determined in patients’ tumors by Western blot. Table S1: Procedure for cDNA reverse transcription synthesis. Table S2: qPCR primer sequence information. Table S3: Main experimental software and network resources qPCR algorithm. Table S4. Fluorescence quantitative PCR reaction SYBR Green system. Table S5: Fluorescence quantitative PCR reaction procedure.

## Data Availability

The data that support the findings of this study are available from the corresponding author upon reasonable request.

## References

[1] Huang J. , Ngai C. H. , and Deng Y. , et al.Cancer Incidence and Mortality in Asian Countries: A Trend Analysis, Cancer Control. (2022) 29, 10.1177/10732748221095955, 10732748221095955. 35770775 PMC9252010

[2] Bray F. , Laversanne M. , and Sung H. , et al.Global Cancer Statistics 2022: GLOBOCAN Estimates of Incidence and Mortality Worldwide for 36 Cancers in 185 Countries, CA: A Cancer Journal for Clinicians. (2024) 74, no. 3, 229–263, 10.3322/caac.21834. 38572751

[3] Feng R.-M. , Zong Y.-N. , Cao S.-M. , and Xu R.-H. , Current Cancer Situation in China: Good or Bad News From the 2018 Global Cancer Statistics?, Cancer Communications. (2019) 39, no. 1, 10.1186/s40880-019-0368-6, 22. 31030667 PMC6487510

[4] Barzaman K. , Karami J. , and Zarei Z. , et al.Breast Cancer: Biology, Biomarkers, and Treatments, International Immunopharmacology. (2020) 84, 10.1016/j.intimp.2020.106535, 106535. 32361569

[5] Liang Y. , Zhang H. , Song X. , and Yang Q. , Metastatic Heterogeneity of Breast Cancer: Molecular Mechanism and Potential Therapeutic Targets, Seminars in Cancer Biology. (2020) 60, 14–27, 10.1016/j.semcancer.2019.08.012. 31421262

[6] Liu S.-Q. , Gao Z.-J. , and Wu J. , et al.Single-Cell and Spatially Resolved Analysis Uncovers Cell Heterogeneity of Breast Cancer, Journal of Hematology & Oncology. (2022) 15, no. 19, 10.1186/s13045-022-01236-0. PMC8895670 35241110

[7] Turner K. M. , Yeo S. K. , Holm T. M. , Shaughnessy E. , and Guan J.-L. , Heterogeneity Within Molecular Subtypes of Breast Cancer, American Journal of Physiology-Cell Physiology. (2021) 321, no. 2, C343–C354, 10.1152/ajpcell.00109.2021. 34191627 PMC8424677

[8] Song G. , Darr D. B. , and Santos C. M. , et al.Effects of Tumor Microenvironment Heterogeneity on Nanoparticle Disposition and Efficacy in Breast Cancer Tumor Models, Clinical Cancer Research. (2014) 20, no. 23, 6083–6095, 10.1158/1078-0432.CCR-14-0493. 25231403 PMC4565518

[9] Kehrberg R. J. , Bhyravbhatla N. , Batra S. K. , and Kumar S. , Epigenetic Regulation of Cancer-Associated Fibroblast Heterogeneity, Biochimica et Biophysica Acta (BBA)–Reviews on Cancer. (2023) 1878, no. 3, 10.1016/j.bbcan.2023.188901, 188901. 37120098 PMC10375465

[10] Liu T. , Lin B. , and Qin J. , Carcinoma-Associated Fibroblasts Promoted Tumor Spheroid Invasion on a Microfluidic 3D Co-Culture Device, Lab on a Chip. (2010) 10, no. 13, 1671–1677, 10.1039/c000022a. 20414488

[11] Mhaidly R. and Mechta-Grigoriou F. , Fibroblast Heterogeneity in Tumor Micro-Environment: Role in Immunosuppression and New Therapies, Seminars in Immunology. (2020) 48, 10.1016/j.smim.2020.101417, 101417. 33077325

[12] Hassona Y. , Cirillo N. , Heesom K. , Parkinson E. K. , and Prime S. S. , Senescent Cancer-Associated Fibroblasts Secrete Active MMP-2 That Promotes Keratinocyte Dis-Cohesion and Invasion, British Journal of Cancer. (2014) 111, no. 6, 1230–1237, 10.1038/bjc.2014.438. 25117810 PMC4453858

[13] Liu Y. , Zhang X. , and Gu W. , et al.Unlocking the Crucial Role of Cancer-Associated Fibroblasts in Tumor Metastasis: Mechanisms and Therapeutic Prospects, Journal of Advanced Research. (2025) 71, 399–413, 10.1016/j.jare.2024.05.031. 38825314 PMC12126706

[14] Cords L. , Tietscher S. , and Anzeneder T. , et al.Cancer-Associated Fibroblast Classification in Single-Cell and Spatial Proteomics Data, Nature Communications. (2023) 14, no. 1, 10.1038/s41467-023-39762-1, 4294. PMC10354071 37463917

[15] Li X. , Sun Z. , and Peng G. , et al.Single-Cell RNA Sequencing Reveals a Pro-Invasive Cancer-Associated Fibroblast Subgroup Associated With Poor Clinical Outcomes in Patients With Gastric Cancer, Theranostics. (2022) 12, no. 2, 620–638, 10.7150/thno.60540. 34976204 PMC8692898

[16] Khaliq A. M. , Erdogan C. , and Kurt Z. , et al.Refining Colorectal Cancer Classification and Clinical Stratification Through a Single-Cell Atlas, Genome Biology. (2022) 23, no. 1, 10.1186/s13059-022-02677-z, 113. 35538548 PMC9092724

[17] Chen B. , Chan W. N. , and Xie F. , et al.The Molecular Classification of Cancer-Associated Fibroblasts on a Pan-Cancer Single-Cell Transcriptional Atlas, Clinical and Translational Medicine. (2023) 13, no. 12, 10.1002/ctm2.1516. PMC10751516 38148640

[18] Kieffer Y. , Hocine H. R. , and Gentric G. , et al.Single-Cell Analysis Reveals Fibroblast Clusters Linked to Immunotherapy Resistance in Cancer, Cancer Discovery. (2020) 10, no. 9, 1330–1351, 10.1158/2159-8290.CD-19-1384. 32434947

[19] Luo W. , Wen T. , and Qu X. , Tumor Immune Microenvironment-Based Therapies in Pancreatic Ductal Adenocarcinoma: Time to Update the Concept, Journal of Experimental & Clinical Cancer Research. (2024) 43, no. 1, 10.1186/s13046-023-02935-3, 8. 38167055 PMC10759657

[20] Peng J. , Sun B.-F. , and Chen C.-Y. , et al.Single-Cell RNA-Seq Highlights Intra-Tumoral Heterogeneity and Malignant Progression in Pancreatic Ductal Adenocarcinoma, Cell Research. (2019) 29, no. 9, 725–738, 10.1038/s41422-019-0195-y. 31273297 PMC6796938

[21] Kochetkova M. and Samuel M. S. , Differentiation of the Tumor Microenvironment: Are CAFs the Organizer?, Trends in Cell Biology. (2022) 32, no. 4, 285–294, 10.1016/j.tcb.2021.11.008. 34895986

[22] Rizzolio S. , Giordano S. , and Corso S. , The Importance of Being CAFs (in Cancer Resistance to Targeted Therapies), Journal of Experimental & Clinical Cancer Research. (2022) 41, no. 1, 10.1186/s13046-022-02524-w, 319. 36324182 PMC9632140

[23] Sulaiman R. , De P. , and Aske J. C. , et al.A CAF-Based Two-Cell Hybrid Co-Culture Model to Test Drug Resistance in Endometrial Cancers, Biomedicines. (2023) 11, no. 5, 10.3390/biomedicines11051326, 1326. 37238998 PMC10216115

[24] Kalinsky K. , Barlow W. E. , and Gralow J. R. , et al.21-Gene Assay to Inform Chemotherapy Benefit in Node-Positive Breast Cancer, New England Journal of Medicine. (2021) 385, no. 25, 2336–2347, 10.1056/NEJMoa2108873. 34914339 PMC9096864

[25] Cardoso F. , Kyriakides S. , and Ohno S. , et al.Early Breast Cancer: ESMO Clinical Practice Guidelines for Diagnosis, Treatment and Follow-Up, Annals of Oncology. (2019) 30, no. 8, 1194–1220, 10.1093/annonc/mdz173. 31161190

[26] Jin M. , Zhang J. , Sun Y. , Liu G. , and Wei X. , ANXA5: Related Mechanisms of Osteogenesis and Additional Biological Functions, Frontiers in Cell and Developmental Biology. (2025) 13, 10.3389/fcell.2025.1553683, 1553683. 40342928 PMC12058784

[27] Wang Z. , Chen H. , and Sun L. , et al.Uncovering the Potential of APOD as a Biomarker in Gastric Cancer: A Retrospective and Multi-Center Study, Computational and Structural Biotechnology Journal. (2024) 23, 1051–1064, 10.1016/j.csbj.2024.02.015. 38455068 PMC10918487

[28] Dong X.-Z. , Zhao Z.-R. , Hu Y. , Lu Y.-P. , Liu P. , and Zhang L. , LncRNA COL1A1-014 is Involved in the Progression of Gastric Cancer via Regulating CXCL12-CXCR4 Axis, Gastric Cancer. (2020) 23, no. 2, 260–272, 10.1007/s10120-019-01011-0. 31650323

[29] Zhou Y. and He M. , GSN Synergies With Actin-Related Transfer Molecular Chain to Promote Invasion and Metastasis of HCC, Clinical & Translational Oncology: Official Publication of the Federation of Spanish Oncology Societies and of the National Cancer Institute of Mexico. (2023) 25, 482–490, 10.1007/s12094-022-02961-1. 36192574 PMC9873781

[30] Zhang Y. , Luo X. , and Lin J. , et al.Gelsolin Promotes Cancer Progression by Regulating Epithelial-Mesenchymal Transition in Hepatocellular Carcinoma and Correlates With a Poor Prognosis, Journal of Oncology. (2020) 2020, 10.1155/2020/1980368, 1980368. 32377190 PMC7199561

[31] Brahmkhatri V. P. , Prasanna C. , and Atreya H. S. , Insulin-Like Growth Factor System in Cancer: Novel Targeted Therapies, BioMed Research International. (2015) 2015, 10.1155/2015/538019, 538019. 25866791 PMC4383470

[32] Rosales-Reynoso M. A. , Rosas-Enríquez V. , and Saucedo-Sariñana A. M. , et al.Genotypes and Haplotypes in the AXIN2 and TCF7L2 Genes are Associated With Susceptibility and With Clinicopathological Characteristics in Breast Cancer Patients, British Journal of Biomedical Science. (2022) 79, 10.3389/bjbs.2021.10211, 10211. 35996498 PMC8915722

[33] Zheng A. , Song X. , and Zhang L. , et al.Long Non-Coding RNA LUCAT1/miR-5582-3p/TCF7L2 Axis Regulates Breast Cancer Stemness Via Wnt/β-Catenin Pathway, Journal of Experimental & Clinical Cancer Research. (2019) 38, no. 1, 10.1186/s13046-019-1315-8, 305. 31300015 PMC6626338

[34] van der Wal T. , Lambooij J.-P. , van Amerongen R. , and Koval M. , TMEM98 is a Negative Regulator of FRAT Mediated Wnt/ß-Catenin Signalling, PLoS ONE. (2020) 15, no. 1, 10.1371/journal.pone.0227435. PMC6974163 31961879

[35] Liao S. , Liu C. , Zhu G. , Wang K. , Yang Y. , and Wang C. , Relationship Between SDC1 and Cadherin Signalling Activation in Cancer, Pathology–Research and Practice. (2020) 216, no. 1, 10.1016/j.prp.2019.152756, 152756. 31810587

[36] Wang Q. , Li D. , and Ma H. , et al.Tumor Cell-Derived EMP1 is Essential for Cancer-Associated Fibroblast Infiltration in Tumor Microenvironment of Triple-Negative Breast Cancer, Cell Death & Disease. (2025) 16, no. 1, 10.1038/s41419-025-07464-9, 143. 40016223 PMC11868485

[37] Cañellas-Socias A. , Cortina C. , and Hernando-Momblona X. , et al.Metastatic Recurrence in Colorectal Cancer Arises From Residual EMP1+ Cells, Nature. (2022) 611, no. 7936, 603–613, 10.1038/s41586-022-05402-9. 36352230 PMC7616986

[38] Cha Y. J. and Koo J. S. , Expression and Role of Epithelial Membrane Proteins in Tumorigenesis of Hormone Receptor-Positive Breast Cancer, Journal of Breast Cancer. (2020) 23, no. 4, 10.4048/jbc.2020.23.e42, 385. 32908789 PMC7462814

[39] Sun G. G. , Wang Y. D. , Lu Y. F. , and Hu W. N. , EMP1, a Member of a New Family of Antiproliferative Genes in Breast Carcinoma, Tumor Biology. (2014) 35, no. 4, 3347–3354, 10.1007/s13277-013-1441-4. 24402572

[40] Zhang H. , Zeng Y. , Ye C. , Cai J. , and Hu X. , Inhibition of FAM114A1 Suppresses Hepatocellular Carcinoma by Targeting AKT1 Signaling, Annals of Clinical and Laboratory Science. (2024) 54, no. 3, 378–387. 39048162

[41] Miao L. , Jiang Z. , and Wang J. , et al.Epithelial Membrane Protein 1 Promotes Glioblastoma Progression Through the PI3K/AKT/mTOR Signaling Pathway, Oncology Reports. (2019) 42, 605–614, 10.3892/or.2019.7204. 31233190 PMC6609345

[42] Jain A. , Jain A. , and Tindell C. A. , et al.Epithelial Membrane Protein-1 Is a Biomarker of Gefitinib Resistance, Proceedings of the National Academy of Sciences of the United States of America. (2005) 102, no. 33, 11858–11863, 10.1073/pnas.0502113102. 16087880 PMC1187965

[43] Wang J. , Li X. , and Wu H. , et al.EMP1 Regulates Cell Proliferation, Migration, and Stemness in Gliomas Through PI3K-AKT Signaling and CD44, Journal of Cellular Biochemistry. (2019) 120, no. 10, 17142–17150, 10.1002/jcb.28974. 31111534

[44] Gu X.-L. , Ou Z.-L. , and Lin F.-J. , et al.Expression of CXCL14 and Its Anticancer Role in Breast Cancer, Breast Cancer Research and Treatment. (2012) 135, no. 3, 725–735, 10.1007/s10549-012-2206-2. 22910931

[45] Gibbs C. , So J. Y. , Ahad A. , Michalowski A. M. , Son D.-S. , and Li Y. , CXCL14 Attenuates Triple-Negative Breast Cancer Progression by Regulating Immune Profiles of the Tumor Microenvironment in a T Cell-Dependent Manner, International Journal of Molecular Sciences. (2022) 23, no. 16, 10.3390/ijms23169314, 9314. 36012586 PMC9409254

[46] Wang Y. , Bi X. , Luo Z. , Wang H. , Ismtula D. , and Guo C. , Gelsolin: A Comprehensive Pan-Cancer Analysis of Potential Prognosis, Diagnostic, and Immune Biomarkers, Frontiers in Genetics. (2023) 14, 10.3389/fgene.2023.1093163, 1093163. 37035750 PMC10076574

[47] Sotgia F. , Martinez-Outschoorn U. E. , and Lisanti M. P. , Mitochondrial Oxidative Stress Drives Tumor Progression and Metastasis: Should We Use Antioxidants as a Key Component of Cancer Treatment and Prevention?, BMC Medicine. (2011) 9, no. 1, 10.1186/1741-7015-9-62, 62. 21605374 PMC3123229

[48] Ran Y. , Wu K. , and Hu C. , et al.Downregulated APOD and FCGR2A Correlates With Immune Infiltration and Lipid-Induced Symptoms of Irritable Bowel Syndrome, Scientific Reports. (2023) 13, no. 1, 10.1038/s41598-023-41004-9, 14211. 37648784 PMC10469184

[49] Gan L. , Lu T. , and Lu Y. , et al.Endosialin-Positive CAFs Promote Hepatocellular Carcinoma Progression by Suppressing CD8+ T Cell Infiltration, Journal for ImmunoTherapy of Cancer. (2024) 12, no. 9, 10.1136/jitc-2024-009111. PMC11535718 39260826

[50] Li Z. , Liang P. , and Chen Z. , et al.CAF-Secreted LOX Promotes PD-L1 Expression Via Histone Lactylation and Regulates Tumor EMT Through TGFβ/IGF1 Signaling in Gastric Cancer-PubMed, Cellular Signalling. (2024) 124, 10.1016/j.cellsig.2024.111462, 111462. 39395525

[51] Shi J. , Feng J. , and Xie J. , et al.Targeted Blockade of TGF-β and IL-6/JAK2/STAT3 Pathways Inhibits Lung Cancer Growth Promoted by Bone Marrow-Derived Myofibroblasts, Scientific Reports. (2017) 7, no. 1, 10.1038/s41598-017-09020-8, 8660. 28819126 PMC5561133

[52] Neal J. T. , Li X. , and Zhu J. , et al.Organoid Modeling of the Tumor Immune Microenvironment, Cell. (2018) 175, no. 7, 1972–1988.e16, 10.1016/j.cell.2018.11.021. 30550791 PMC6656687

[53] Chen P.-Y. , Wei W.-F. , Wu H.-Z. , Fan L.-S. , and Wang W. , Cancer-Associated Fibroblast Heterogeneity: A Factor That Cannot be Ignored in Immune Microenvironment Remodeling, Frontiers in Immunology. (2021) 12, 10.3389/fimmu.2021.671595, 671595. 34305902 PMC8297463

[54] Mao X. , Xu J. , and Wang W. , et al.Crosstalk Between Cancer-Associated Fibroblasts and Immune Cells in the Tumor Microenvironment: New Findings and Future Perspectives, Molecular Cancer. (2021) 20, no. 1, 10.1186/s12943-021-01428-1, 131. 34635121 PMC8504100

