# Supplementary material for: A Novel CAF‐Related Signature for Precise Prediction of Clinical Outcomes and Immunotherapy Response for Breast Cancer Patients: Based on Multiomics Analyses and Experimental Validation
Source: Mediators Inflamm. 2026 Jun 26;2026:9934495. doi: 10.1155/mi/9934495 (PMC13306116; doi:10.1155/mi/9934495)
Supplement: Supplementary file 1 — Supporting Information Figure S1: The proportion of immune cells across groups was calculated using EPIC. Figure S2: XCELL calculated immune cell ratios between different groups. Figure S3: CAFs’ risk factors are negatively correlated with the level of CD8+ immune cell infiltration. The expression was determined in patients’ tumors by Western blot. Table S1: Procedure for cDNA reverse transcription synthesis. Table S2: qPCR primer sequence information. Table S3: Main experimental software and network resources qPCR algorithm. Table S4. Fluorescence quantitative PCR reaction SYBR Green system. Table S5: Fluorescence quantitative PCR reaction procedure. [file MI-2026-9934495-s001.docx]

**Materials and methods**

**qPCR mRNA analysis**

Extraction of RNA from formalin-fixed, paraffin-embedded (FFPE) tissues was done as follows. 5-10 µm-thin sections were deposited into a 1.5 ml microcentrifuge tube, and 1 ml of xylene was added, mixed thoroughly, and then removed. The above mixture was incubated at room temperature for 5 minutes and then centrifuged for 2 minutes at 12,000 rpm. The deparaffinization procedure was repeated to obliterate paraffin. Next, 1 ml of 100% ethanol was added to the xylene. After thorough mixing, the upper layer was separated and discarded. Several ethanol washes were performed to maintain tissue purity. Finally, the tissues were allowed to air dry at room temperature.

A proper amount of Trizol lysate was added to the tissues. The tissues were then mixed until smooth. The liquid was transferred to a new 1.5 mL tube and placed in a 55 °C water bath for 15 minutes. This step broke the link between RNA and proteins. Chloroform was then added to ensure complete homogenization, and the mixture was allowed to stand for 10 minutes. This was followed by centrifugation at 14,000 rpm for 15 minutes, after which the water molecules from the previous step were carefully transferred to a new centrifuge tube to prevent them from being absorbed.

The same volume of pre-cooled isopropanol was then injected into the centrifuge tubes containing the water, and the mixture was stored overnight at -20℃ or left at room temperature for 10 minutes before centrifuging the RNA to precipitate. The precipitate was then washed with 75% ethanol; the top layer of liquid was removed by centrifugation, and it was finally dried. We added DEPC water to dissolve the RNA. The RNA concentration was measured with a UV spectrophotometer. The OD 260/280 ratio was maintained between 1.8 and 2.0 to ensure the RNA was of good quality. Procedure for cDNA reverse transcription synthesis: 500 ng of RNA sample was selected, and the reaction system was set up to remove genomic DNA according to the instructions below.

Table S1. Procedure for cDNA reverse transcription synthesis.

| reactant | volume (μL) |
| --- | --- |
| 2×5×gDNA Eraser buffer | 2 |
| 1× gDNA Eraser | 1 |
| 6×RNAse-free water | up to 10μl |
| RNA | 1.0 μg |

The reaction procedure is as follows:

Step1: 42°C 2 min

Step2: 4°C forever

Once the genomic DNA has been removed, we set up the reverse transcription reaction system according to the table and add it to the previous genomic DNA reaction system.

Table S2. qPCR primer sequence information

| Lot No | Oligo Name | Sequence (5 to 3) |
| --- | --- | --- |
| 2215467488 | GAPDH FORWARD | CAA ATT CCA TGG CAC CGT CAA G |
| 2215467489 | GAPDH REVERSE | ATC GCC CCA CTT GAT TTT GGA G |
| 2215467490 | TMEM98 FORWARD | ATG TCC CAC TGC ATT GCC ATC |
| 2215467491 | TMEM98 REVERSE | ACC ACA ATG ATG TCG CTG ACA C |
| 2215467492 | TCF7L2 FORWARD | AAA GTG CGT TCG CTA CAT ACA A |
| 2215467493 | TCF7L2 REVERSE | GAG GCG AAT CTA GTA AGC TTC C |
| 2215467494 | SDC1 FORWARD | AAG ATA TCA CCT TGT CAC AGC A |
| 2215467495 | SDC1 REVERSE | GTT CTG GAG ACG TGG GAA TAG |
| 2215467496 | PPIB FORWARD | TTC TTC ATC ACG ACA GTC AAG A |
| 2215467497 | PPIB REVERSE | TCA CAT CCT TCA GGG GTT TAT C |
| 2215467498 | IGFBP4 FORWARD | AGA AGC ACT TCG CCA AAA TTC |
| 2215467499 | IGFBP4 REVERSE | GAT GAT GTA GAG GTC CTC GTG |
| 2215467500 | GSN FORWARD | CAG AGA CTC TTC CAG GTC AAA G |
| 2215467501 | GSN REVERSE | CAG TCT TTC ATA CCG ATT GCT G |
| 2215467502 | FAM114A1 FORWARD | TGG AGA CAA AGC AGA AGT TAC T |
| 2215467503 | FAM114A1 REVERSE | ACA TTC AGC AAG TGT ATC CTC A |
| 2215467504 | EMP1 FORWARD | CAT TAT GCG AAT CGT GAT GGA A |
| 2215467505 | EMP1 REVERSE | GAT GAT GAA GCT GAA GCA GAA G |
| 2215467506 | CXCL14 FORWARD | CTG GAA ATG AAG CCA AAG TAC C |
| 2215467507 | CXCL14 REVERSE | GTT GTA CCA CTT GAT GAA GCG |
| 2215467508 | APOD FORWARD | GCT GGA AGT TAA GTT TTC CTG G |
| 2215467509 | APOD REVERSE | GGA TGA TGC AGG TAC AGG AAT A |
| 2215467510 | ANXA5 FORWARD | AAC TCT TCG GAA GGC TAT GAA A |
| 2215467511 | ANXA5 REVERSE | TGC CAA ACA GAG TCT TAA AAG C |

Table S3. Main experimental software and network resources qPCR algorithm (relative quantification, 2^-ΔΔCt^law)

| software | edition | Developer or URL |
| --- | --- | --- |
| Primer Premier | Version 6 | Premier Biosoft Inc., CA |
| Oligo | Version 7.37 | Molecular Biology Insights Inc., USA |
| Bio-Rad CFX Manager | Version 3.1 | Bio-Rad, USA |
| SPSS | Version 19 | International Business Machines Inc., USA |
| sigmaplot | Version 12.3 | Systat Software Inc., USA |
| NCBI | / | http://www.ncbi.nlm.nih.gov/ |

Table S4. Fluorescence quantitative PCR reaction SYBR Green system (10 μL)

| reactant | volume (μl) |
| --- | --- |
| 2×SYBR Green Mix | 5ul |
| Primer_F+R(each 10uM) | each 0.5ul |
| cDNA | 2ul |
| ddH2O | to 10ul |

Table S5. Fluorescence quantitative PCR reaction procedure

| project | temperature | time | recurring number |
| --- | --- | --- | --- |
| Pre-oxidation | 95℃ | 5min | 1 |
| denaturation | 95℃ | 10sec | 44 |
| Annealing/extension | 60℃ | 30sec |  |
| Melting curve collection | 95℃ | 15sec | 1 |
|  | 60℃ | 60sec |  |
|  | 95℃ | 30sec |  |
|  | 95℃ | 15sec |  |

**Supplementary figures:**

**CAFs' risk factors in the tumor microenvironment are correlated with immune cell infiltration**

EPIC and XCELL analyzed immune infiltration in the samples, and significant differences were observed across multiple immune cell types between CAF-related signature groups (Figure S1, Figure S2).


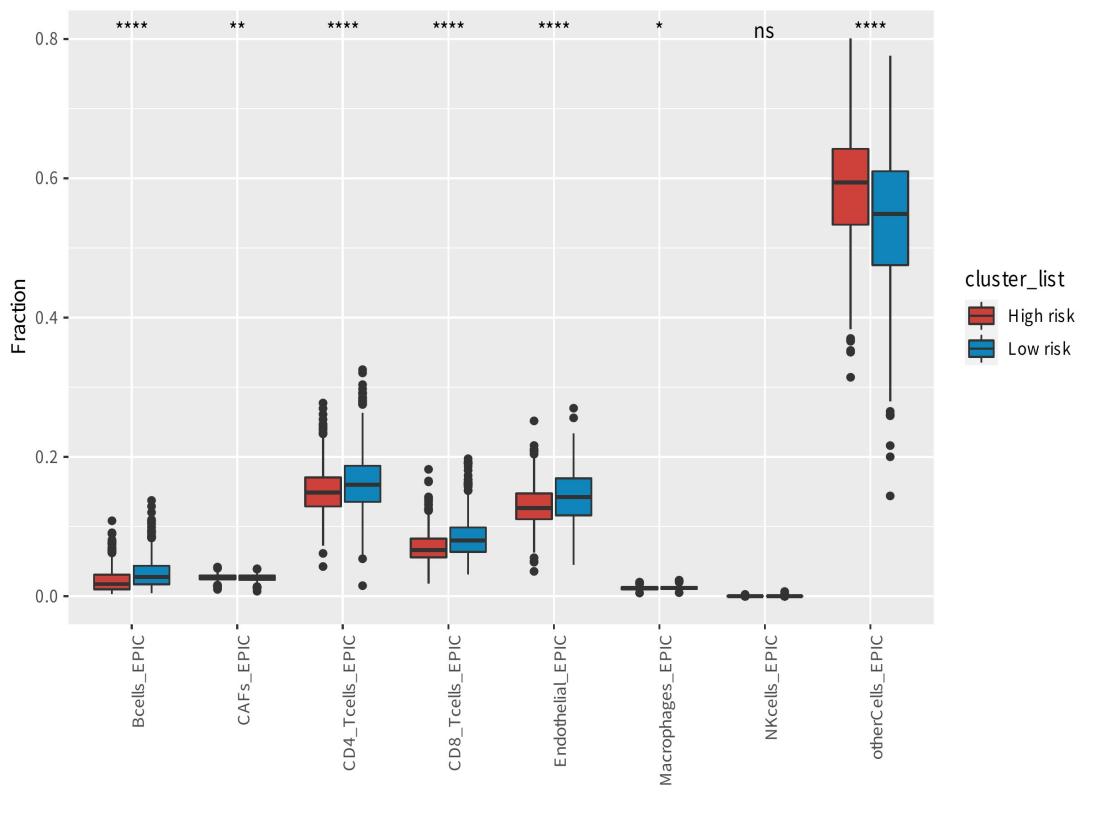


Figure S1. The proportion of immune cells across groups was calculated using EPIC.


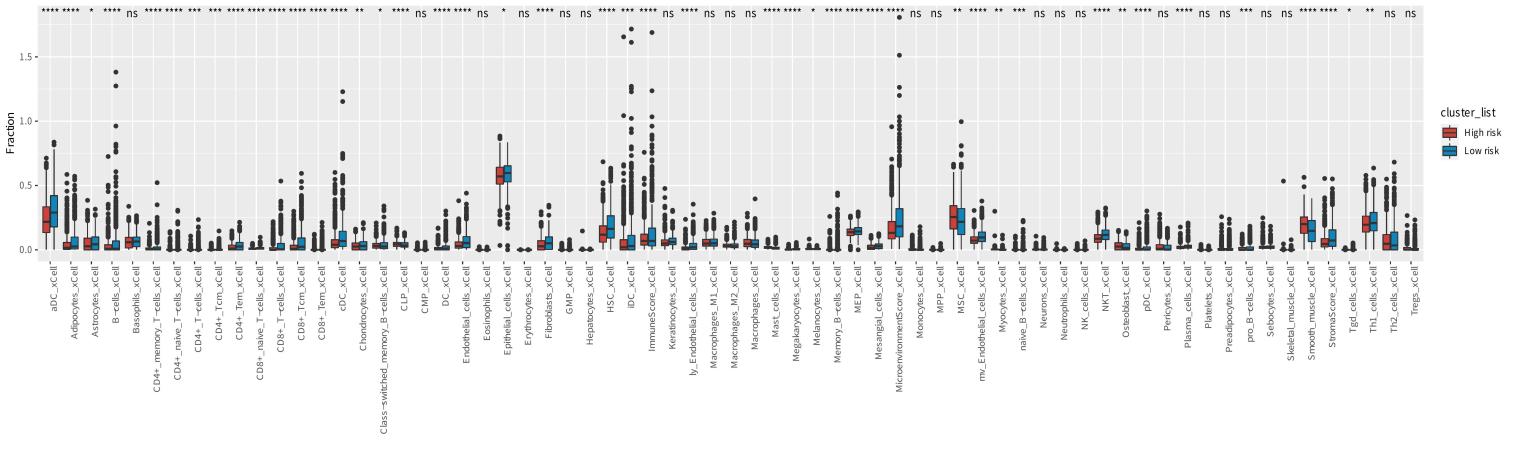
Figure S2. XCELL calculated immune cell ratios between different groups.

Intraoperative frozen fresh tumor tissues were obtained from clinical patients, and the expression level of the high-risk factor EMP1 in breast cancer tissues of the high-risk and low-risk groups with CAFs characteristics was detected by Western blot (n = 6, technical repetition 3 times). Therefore, the protein expressions of high-risk factors EMP1 and αSMA in the tissues of patients in the high-risk CAFs group were significantly higher than those in the low-risk CAFs group, and the protein expression of CD8+ T cells was considerably lower than that in the low-risk CAFs group (Figure S3).


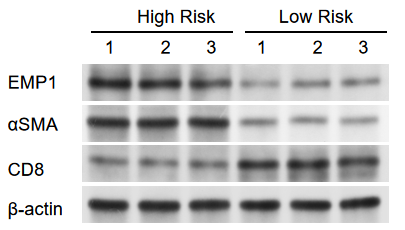


Figure S3: CAFs' risk factors are negatively correlated with the level of CD8+ immune cell infiltration. The expression was determined in patients' tumors by Western blot.
